# Effect of food-related behavioral activation therapy on food intake and the environmental impact of the diet: results from the MooDFOOD prevention trial

**DOI:** 10.1007/s00394-019-02106-1

**Published:** 2019-10-23

**Authors:** Alessandra C. Grasso, Margreet R. Olthof, Corné van Dooren, Miquel Roca, Margalida Gili, Marjolein Visser, Mieke Cabout, Mariska Bot, Brenda W. J. H. Penninx, Gerard van Grootheest, Elisabeth Kohls, Ulrich Hegerl, Matthew Owens, Ed Watkins, Ingeborg A. Brouwer, the MooDFOOD Prevention Trial Investigators, Marjolein Visser, Ingeborg A. Brouwer, Mieke Cabout, Brenda Penninx, Mariska Bot, Nadine Paans, Carisha Thesing, Deborah Gibson-Smith, Melany Horsfall, Lena Weiss, Ed Watkins, Matthew Owens, Amy Romijn, Hannah Bunce, Owain Winfield, Miquel Roca, Margarita Gili, Miquel Tortella, Clara Homar Covas, Margalida Vives Forteza, Adoración Castro Gracia, Maria Angeles Pérez-Ara, José Luis Reig, Ulrich Hegerl, Elisabeth Kohls, Jana Hoesel, Ezgi Dogan, Sabrina Baldofski, Nicole Mauche, Brenda Penninx, Gerard van Grootheest, Bep Verkerk

**Affiliations:** 1 Department of Health Sciences, Faculty of Science, and Amsterdam Public Health Research Institute, Vrije Universiteit (VU) Amsterdam, De Boelelaan 1085, 1081 HV Amsterdam, The Netherlands; 2 Netherlands Nutrition Centre (Voedingscentrum), Bezuidenhoutseweg 105, 2594 AC The Hague, The Netherlands; 3 Institut Universitari d’Investigació en Ciències de la Salut (IUNICS/IDISBA), Rediapp, University of Balearic Islands, Carretera De Valldemossa km 7.5, 07122 Palma de Mallorca, Spain; 4 Department of Psychiatry, Amsterdam Public Health Research Institute, and GGZ inGeest Specialized Mental Health Care, Amsterdam UMC, VU Amsterdam, De Boelelaan 1117, 1081 HV Amsterdam, The Netherlands; 5 Department of Psychiatry and Psychotherapy, Medical Faculty, University Leipzig, Semmelweisstr. 10, Haus 13, 04103 Leipzig, Germany; 6 Department of Psychiatry, Psychosomatics and Psychotherapy, Medical Faculty, Goethe-University Frankfurt, Heinrich-Hoffmann-Str. 10, 60528 Frankfurt a.M., Germany; 7 Department of Psychology, University of Exeter, Perry Road, Exeter, EX4 4QG UK

**Keywords:** Sustainability, Diet, RCT, Depression

## Abstract

**Purpose:**

Food-based dietary guidelines are proposed to not only improve diet quality, but to also reduce the environmental impact of diets. The aim of our study was to investigate whether food-related behavioral activation therapy (F-BA) applying Mediterranean-style dietary guidelines altered food intake and the environmental impact of the diet in overweight adults with subsyndromal symptoms of depression.

**Methods:**

In total 744 adults who either received the F-BA intervention (F-BA group) or no intervention (control group) for 12 months were included in this analysis. Food intake data were collected through a food frequency questionnaire at baseline and after 6 and 12 months. Greenhouse gas emissions (GHGE), land use (LU), and fossil energy use (FEU) estimates from life-cycle assessments and a weighted score of the three (*p*ReCiPe score) were used to estimate the environmental impact of each individual diet at each timepoint.

**Results:**

The F-BA group reported increased intakes of vegetables (19.7 g/day; 95% CI 7.8–31.6), fruit (23.0 g/day; 9.4–36.6), fish (7.6 g/day; 4.6–10.6), pulses/legumes (4.0 g/day; 1.6–6.5) and whole grains (12.7 g/day; 8.0–17.5), and decreased intake of sweets/extras (− 6.8 g/day; − 10.9 to − 2.8) relative to control group. This effect on food intake resulted in no change in GHGE, LU, and *p*ReCiPe score, but a relative increase in FEU by 1.6 MJ/day (0.8, 2.4).

**Conclusions:**

A shift towards a healthier Mediterranean-style diet does not necessarily result in a diet with reduced environmental impact in a real-life setting.

**Trial registration:**

ClinicalTrials.gov. Number of identification: NCT02529423. August 2015.

**Electronic supplementary material:**

The online version of this article (10.1007/s00394-019-02106-1) contains supplementary material, which is available to authorized users.

## Introduction

A transition from traditional to current dietary patterns has contributed to a rise in global prevalence of chronic diseases and to unprecedented changes in ecosystems, both of which are threatening public health [1]. Food production is largely responsible for the environmental burdens associated with the human diet, including climate change, biodiversity loss, and pollution [2], with the other stages in the supply chain (i.e., processing, distribution, retailing, home food preparation, and waste) playing a part. Food production contributes to approximately 16–25% of total global greenhouse gas emissions (GHGE) [3], and it is estimated that this will increase by 51% from 2005/07 to 2050 if dietary patterns do not change [4]. Currently croplands and pastures cover 37% of total land area [5], making agriculture the largest use of land on the planet. It is estimated that food production will need to increase by 25–70% to meet 2050 food demand [6]. While sustainable intensification of agriculture is proposed as a solution to increase food production with reduced environmental risks, it will not prevent further agricultural expansion driven by the projected demand [7]. Thus, dietary change has been identified as an essential counterpart to reduce the environmental pressures associated with the diet and to provide food security for future generations [8–10].

Recent research has increasingly focused on evaluating the environmental impact of habitual dietary choices, predefined diets, and alternative dietary patterns to propose more sustainable dietary patterns [1, 11–13]. An assortment of sustainable diets has been proposed, such as vegan and Mediterranean, as well as following national food-based dietary recommendations. Environmental as well as health benefits of these diets have been attributed to partial substitution of animal-based foods with plant-based foods [1, 12, 14, 15], and also to reduced caloric intake [16–19]. While these studies have predominately examined the environmental impact of hypothetical change from current to proposed diets, there is limited research on the environmental impact of dietary change in a real-life setting. Only one previous study has examined changes in GHGE related to changes in food choice in overweight women who received a diet plan based on the Nordic Nutrition Recommendations 2004 and found no effect on diet-associated GHGE, although the women increased their fruit and vegetable intake and decreased total caloric intake compared to those who did not receive the diet plan [20]. Thus, the environmental impact of dietary change in line with dietary guidelines in a real-life setting needs further investigation.

In the recent MooDFOOD (Multi-country cOllaborative project on the rOle of Diet, FOod-related behavior, and Obesity in the prevention of Depression) trial [21], 1025 overweight adults aged 18–75 years with subsyndromal symptoms of depression were randomized to a 12-month food-related behavioral activation therapy (F-BA) intervention (F-BA group) and were provided with dietary guidelines based on a Mediterranean-style diet (Table 1), or to a control group that received no F-BA intervention. Although the F-BA intervention was designed to change diet and behavior to prevent the onset of depression, we hypothesized that the F-BA intervention would improve the environmental sustainability of the diet, as it focused on shifting habitual eating patterns to a Mediterranean-style diet [22]. Therefore, this study aimed to investigate whether the F-BA intervention changed food intake and to assess the environmental impact of the observed dietary change.

**Table 1:** MooDFOOD dietary guidelines

Food group	Guideline
Vegetables *Examples*^a^: green leafy and salad vegetables, fruit vegetables (e.g., cucumber and courgette), flower and flower buds (e.g., broccoli), bulb and stem vegetables (e.g., onion); root and tubers; sea vegetables. Excludes potatoes	300–400 g/day
Fruit *Examples*: core fruit, stone fruit, berries, citrus fruits, tropical fruits, dried fruit	2–3 pieces/day
Fish *Examples*: freshwater fish, salt water fish, white fish, oily fish, shell fish, sustainable fish	3 times/week
Meat *Examples of good meat*: chicken, turkey *Examples of protein*-*rich alternatives:* eggs, nuts, soy products like tofu, fish	Reduce to 300 g/week
Pulses or legumes *Examples*: soy beans, peanuts, fresh peas/beans, dried beans/peas, chickpeas, lentils	3 times/week
Whole grain products *Examples*: whole grain pasta and bread, brown rice, oatmeal, muesli, couscous	Choose
Low-fat dairy products *Examples*: low-fat milk and yogurt, mature cheese, fresh cheese, soy products, cottage cheese	3 servings/day
Olive oil *Examples of use*: in frying food, tossed vegetables, salads, pasta sauces	Use as principal source for cooking
Processed foods and soft drinks *Examples*: (frozen) ready-to-eat meals, processed sandwich meats, sausages, savory snacks, sweet snacks, fried food, sugar-sweetened beverages, sugar added to coffee/tea, fruit juice *Examples of healthy alternatives*: fruit, vegetables, nuts, fish, water, tea or coffee	Limit
Alcoholic beverages *Moderate consumption defined as:* for men, maximum 2 standard glass per day; for women, maximum of 1 standard glass per day	Drink in moderation

MooDFOOD dietary guidelines were based on a Mediterranean-style dietary pattern and provided in the food-related behavioral activation therapy (F-BA) intervention during the 12-month MooDFOOD depression prevention trial. Guidelines were provided orally and in the form of a pamphlet to the intervention participants [21]

^a^Examples of all food groups were provided with pictures along with practical tips to achieve the guideline

## Methods

### Study design and subjects

The MooDFOOD trial was a 12-month randomized controlled prevention trial that investigated the feasibility and effectiveness of two different nutritional strategies for the prevention of depression: multi-nutrient supplementation and F-BA. The design, methods, and primary outcomes of the trial are described in detail elsewhere [21, 23] and are summarized below. A sample of 1025 adults aged 18–75 years with a body mass index (BMI) of 25–40 kg/m^2^ and elevated symptoms for depression (Patient Health Questionnaire-9 score ≥ 5) [24] were recruited from The Netherlands, United Kingdom, Germany, and Spain and randomized to one of the four trial arms according to a 2 × 2 factorial design: (1) multi-nutrient supplement with F-BA intervention (*n* = 256); (2) placebo supplement with F-BA intervention (*n* = 256); (3) multi-nutrient supplement without F-BA intervention (*n* = 256); or (4) placebo supplement without F-BA intervention (*n* = 257). Randomization was stratified according to recruitment site (i.e., country) and participants’ history of depression status at the baseline assessment. Participants, therapists, and researchers were blind to supplement allocation, and researchers were blind to behavioral intervention status when conducting analyses. The four trial arms were condensed to two trial arms to make comparisons in food intake between participants who received the F-BA intervention (F-BA group) and participants who did not receive the F-BA intervention (control group). We assumed that the multi-nutrient supplement had a null effect on food intake and thus was not a focus in this study. We confirmed this by adding supplement status to the statistical models when analyzing intervention effect and it did not affect results.

### F-BA intervention

The F-BA intervention consisted of up to 21 therapy sessions, of which up to 15 were individual sessions and up to 6 were group sessions. The individual sessions were provided in single 30-min or double 1-h meetings occurring at first weekly and then every 2 weeks, while the group sessions included up to 10 people and lasted about 1 h, occurring at first monthly and then bimonthly. Among the 512 participants randomized to the F-BA group, 71% attended at least 8 out of the 21 sessions and were considered compliant (this cutoff for compliance is described by Bot et al. [23]). Participants attended a median of 14 out of 15 individual sessions [interquartile range (IQR) 6–15] and a median of 0 out of 6 group sessions (IQR 0–4) [23]. The control group received no F-BA intervention (*n* = 513 participants).

The F-BA intervention focused on changing food-related behaviors and shifting habitual dietary patterns to improve diet to prevent the onset of depressive episodes; environmental impact of diet was not considered in the design of the intervention. The F-BA intervention incorporated standard approaches of behavioral activation, which focuses on reducing avoidant behaviors and building routines and behaviors that are rewarding and/or pleasant, proven effective in the treatment of depression [25]. Psychologists familiar with behavioral activation were trained and delivered the F-BA intervention under supervision of a dietician. The psychologists helped participants to set goals on introducing healthy foods into their diets as well as reducing consumption of foods considered to be eaten in excess, taking into account baseline records. Goals were revisited and modified when necessary during subsequent sessions. During the intervention, participants kept a record of daily activities and habits, and were able to take notes about their mood and foods eaten during the day. The records aimed to help in the identification of triggers to habits and engagement in self-monitoring to improve food-related behaviors (e.g., regular meals per day, less snacking) and habitual dietary patterns. The participants were provided with a participant manual with detailed information about what was discussed.

### MooDFOOD dietary guidelines

An introduction to healthy eating associated with mood improvement was provided in the third therapy session, which involved the provision of dietary guidelines based on a Mediterranean-style dietary pattern, referred to as the MooDFOOD dietary guidelines (Table 1). The Mediterranean diet served as the basis for the guidelines, because evidence indicates that following such a dietary pattern may prevent the onset of depression [26–31]. The guidelines were adjusted to be more consistent with the national dietary recommendations of the MooDFOOD prevention trial sites [32–36]. The MooDFOOD dietary guidelines consisted of general advice (e.g., limit meat intake to 300 g/week) and more detailed recommendations, and presented examples of ‘good’ and ‘bad’ food choices as well as food exchanges, for example, increase vegetable intake by decreasing intake of potatoes, rice, and/or bread; replace sugared drinks and sweet snacks by fruit; and replace processed sandwich meats by other sandwich toppings such as low-fat cheese, hummus, egg, and fish. No total calorie restriction was advised. In addition to the MooDFOOD dietary guidelines, a description of the link between diet and depression was provided in the F-BA participant manual, with greatest emphasis on the association between consumption of sweets, cakes, pastries, and fast foods and increased risk of depression and consumption of fruit, vegetables, fish, and whole grains and decreased risk of depression. Other foods in the MooDFOOD dietary guidelines such as low-fat dairy, meat, pulses/legumes, and olive oil were only described as part of a healthy diet, and no direct linkage between these foods and depression was made in the manual.

### Dietary data

Participants reported their usual food intake during the previous month by completing an online self-administered food frequency questionnaire (FFQ) at baseline (T0), 6 months (T6) and at 12 months (T12; end of trial). The FFQ was based on the validated GA2LEN FFQ, as it showed to be an appropriate tool to estimate food intake across Europe regardless of cultural and linguistic differences [37]. The FFQ included 210 food items which were categorized into 18 food groups based on food groups for which dietary recommendations were made in the F-BA intervention: (1) vegetables, (2) fruit, (3) fish, (4) meat, (5) egg/soy, (6) pulses/legumes, (7) nuts, (8) potatoes, (9) whole grains, (10) refined grains, (11) low-fat dairy products, (12) high-fat dairy products, (13) olive oil, (14) other fats/oils, (15) sweets/extras, (16) soft drinks (including fruit juices), (17) alcoholic beverages, and (18) water/coffee/tea (see Online Resource 1, Table 1 for FFQ food items and corresponding food group classification). Standard portion sizes following the Food Standard Agency Food Portion Sizes Guidelines were used [38]. Consumption frequency and portion size data were linked with food composition data from the McCance and Widdowson’s composition of foods data set (2015) to calculate total energy intake in kilocalories (kcal) per gram (g) [39]. The percentage of total energy intake (*E*%) contributed by each food group was calculated as the food group energy intake divided by the total energy intake. Food intake was considered missing if a participant completed < 15% of the FFQ. Among the 1025 participants randomized in the MooDFOOD depression prevention trial, 86 had missing dietary data at T0 and 186 had missing dietary data at both follow-up measurements and were excluded from this study. In addition, individuals who under-/over-reported caloric intake were excluded from the analysis. Energy under-/over-reporting was classified as an energy intake spanning above or under the mean plus/minus three standard deviations (sd). Median intakes are reported along with the 25th and 75th percentiles.

### Environmental data

Various measures were investigated to estimate the environmental impact of the diet, namely, GHGE expressed in kilograms of carbon dioxide equivalents (kg CO_2_-eq), land use (LU) in square meter-year (m^2^*y), fossil energy use (FEU) in mega joules (MJ), and a weighted score of the three (*p*ReCiPe score) [40]. GHGE, LU, and FEU were used as indictors due to their availability in reliable data sets and their frequent application in studies examining the environmental impact of diets [41]. Environmental impacts were calculated per 100 g food with life cycle assessments (LCA) from cradle-to-grave by Blonk Consultants (Gouda, The Netherlands) using ReCiPe 2016 Midpoint v1.00 method [42]. LCA is a methodological framework for assessing the environmental impacts over the entire life cycle of a product, from cultivation to packing, consumption and final disposal [43]. For each food item, GHGE, LU, and FEU data were obtained either from an LCA database containing 94 commonly eaten food products based on European Food Safety Authority’s Comprehensive European Food Consumption Database (PROMISS data set 2017) [44] or a database containing 207 commonly eaten food products in The Netherlands, based on the Dutch Consumption Survey 2007–2010 (Optimeal data set 2015) [45]. A weighted combination of GHGE, LU, and FEU was used to calculate a *p*ReCiPe score, a simplified environmental impact score, adapted from the *p*ReCiPe score developed by Tyszler et al. [40]. The *p*ReCiPe score of each food item was calculated by

$$p{\text{ReCiPe}}_{i} = 0.0459 \times {\text{GHGE}}_{i} + 0.0439 \times {\text{LU}}_{i} + 0.0025 \times {\text{FEU}}_{i} ,$$

where $i$ is a food item and GHGE is expressed in kg CO_2_-eq/100 g, LU in m^2^*y/100 g, and FEU in MJ/100 g. This calculation is based on the ReCiPe method which aggregates several LCA impact categories, such as eutrophication and land transformation [46]. The *p*ReCiPe score only includes three of the 16 environmental impact categories (i.e., GHGE, LU and FEU), as they were found to have the most weight in the end score in LCAs of agricultural products [15, 41, 47]. Data were expressed per 100 g food and were used to estimate the overall GHGE, LU, FEU, and *p*ReCiPe score for each individual diet. Environmental data sources and values are available in Online Resource 2.

### Statistical analysis

To analyze the difference in change in food intake and in environmental impact of the diet from T0 to T12 between the F-BA and control groups, longitudinal analysis of covariance using mixed model analysis was used. Participants with missing dietary data at T0 were excluded in the analyses, as the baseline value of the outcome variable was included as a covariate, as well as participants with missing dietary data at both T6 and T12 as individuals with only a baseline measurement are not part of the analysis [48]. Those with missing dietary data at either T6 or T12 were included in the analysis and no imputations were conducted, as mixed model analysis estimated with the maximum likelihood estimator accounts for missing data [49]. In addition to baseline outcome values, adjustment was made for sex (male or female), age (years, continuous), and site (added as another level to the model) for all outcomes. To assess the difference in change in environmental impact of the diet due to change in diet composition between the F-BA and control groups, adjustment for total caloric intake in kcal/day (continuous, time-dependent) was applied. However, since the environmental impact associated with the diet is influenced not only by diet composition, but also caloric quantity [17], the main environmental results presented do not control for caloric intake. To avoid the increased risk of type I error due to multiple testing of the 18 food groups, Holm–Bonferroni correction of the *P* value was done [50]. This procedure is a sequential approach taking into account the total number of hypotheses (18 for 18 food groups), and original *P* values, so that the corrected *P* value for the *i*th test is computed as *P*_Holm–Bonferroni_ = (18 − *i *+ 1)*←*§*P* [51]. This was tested in order from the smallest to largest *P* value and stopped when the first non-significant *P* value was observed based on a 0.05 *α* level. While original *P* values are reported along with 95% confidence intervals (CI), statistical significance is determined by the Holm–Bonferonni adjusted *P* value. The analyses were performed on an intention-to-treat (ITT) basis. Data were analyzed using Stata version 14.2 (StataCorp, TX, USA).

A post hoc per protocol (PP) analysis was done to examine whether those who were more compliant to the F-BA intervention had a greater change in diet and environmental impact of the diet. The cutoff for compliance was attending at least 8 out of the 21 sessions [23]. The same methods were used as in the ITT analysis, but the PP analyses measured the difference in change in food intake and environmental impact of the diet between a subgroup of compliant persons in the F-BA group and the control group over the 12-month period.

## Results

### Study participants and baseline characteristics

In total, 753 participants randomized in the MooDFOOD depression prevention trial had dietary data at T0 and at T6 or T12. The baseline mean ± standard deviation of total caloric intake was 2483.9 ± 2269.9 kcal/day for men and 2347.2 ± 1245.9 kcal/day for women in both groups, resulting in an upper cutoff for implausible caloric intake of 9293.71 kcal/day and 6084.78 kcal/day, respectively. This led to the exclusion of 9 participants due to over-reporting caloric intake at either T0 or at both T6 and T12. Therefore, a total of 744 participants were included in the analyses measuring the intervention effect (flow diagram is available as Online Resource 1, Fig. 1). In general, baseline characteristics of those included in the analysis and those excluded from the analysis were comparable (Online Resource 1, Table 2).

**Table 2:** Baseline characteristics of participants who received food-related behavioral activation therapy (F-BA) intervention (F-BA group) and participants who did not receive the F-BA intervention (control group) (*N* = 744)

Characteristic	F-BA group *n* = 373	Control group *n* = 371
Sex^a^		
Female	78.3 (292)	72.5 (269)
Male	21.7 (81)	27.5 (102)
Age (years)^b^	47.9 + 12.6	47.2 + 13.4
Education^a^		
Low	8.6 (32)	10.8 (40)
Middle	47.5 (177)	46.9 (174)
High	44.0 (164)	42.3 (157)
Site^a^		
Germany	29.0 (108)	31.8 (118)
United Kingdom	24.1 (90)	25.3 (94)
Spain	20.6 (77)	22.1 (82)
The Netherlands	26.3 (98)	20.8 (77)
History of depression^a^		
Yes	31.1 (116)	33.4 (124)
No	68.9 (257)	66.6 (247)
Supplement status^a^		
Multi-nutrient supplement	47.5 (177)	49.1 (182)
Placebo	52.5 (196)	50.9 (189)
BMI (kg/m^2^)^b^	31.2 ± 3.8	31.2 ± 4.1
Total energy intake (kcal/day)^c^	2167.8 (1689.6; 2632.7)	2155.0 (1701.6; 2701.7)
Food intake (g/day)^c^		
Vegetables	292.0 (181.6; 437.1)	302.5 (219.6; 456.2)
Fruit	255.9 (166.7; 412.6)	260.0 (165.7; 448.6)
Fish	44.3 (27.1; 74.9)	42.9 (24.3; 70.0)
Meat	122.4 (79.3; 185.6)	135.0 (86.1; 201.2)
Egg/soy	25.0 (10.7; 42.9)	27.9 (14.3; 49.4)
Pulses/legumes	37.9 (22.5; 62.1)	35.7 (21.8; 62.9)
Nuts	2.1 (0.7; 5.0)	2.1 (0.7; 5.0)
Potatoes	24.5 (13.9; 43.6)	24.3 (12.1; 46.5)
Whole grains	90.6 (46.5; 156.6)	92.8 (43.6; 170.4)
Refined grains	98.6 (62.6; 166.4)	100.4 (57.1; 163.3)
Low-fat dairy	120.0 (17.1; 220.0)	97.1 (17.1; 237.1)
High-fat dairy	89.3 (39.3; 160.4)	94.3 (46.1; 175.4)
Olive oil	9.4 (3.1; 17.3)	8.6 (3.1; 13.4)
Other fats/oils	15.1 (8.0; 27.5)	14.8 (7.1; 27.1)
Sweets/extras	117.0 (70.3; 187.3)	125.2 (77.9; 189.9)
Soft drinks	85.7 (28.6; 197.1)	68.6 (25.7; 200.0)
Alcoholic beverages	42.9 (17.9; 114.3)	45.4 (8.9; 119.6)
Water/coffee/tea	1314.3 (971.4; 1657.1)	1300.0 (914.3; 1700.0)
Environmental indicators^c^		
GHGE^d^ (kg CO_2_-eq/day)	5.73 (4.47; 7.44)	5.94 (4.50; 7.84)
LU^e^ (m^2^*y/day)	4.51 (3.49; 5.81)	4.61 (3.49; 6.12)
FEU^f^ (MJ/day)	40.33 (31.89; 52.35)	41.28 (33.09; 56.95)
*p*ReCiPe score^g^ (points/day)	0.55 (0.44; 0.74)	0.58 (0.45; 0.77)

^a^Values displayed as percentage (frequency)

^b^Values displayed as mean ± SD

^c^Values displayed as median with interquartile range (25; 75th percentile)

^d^Greenhouse gas emission

^e^Land use

^f^Fossil energy use

^g^Weighted average of GHGE, LU and FEU

Similar baseline characteristics of the two study groups were found and are presented in Table 2. The majority of the study participants were female (75.4%) with a mean age of 47.6 years and BMI of 31.2 kg/m^2^. The baseline median total caloric intake of the study participants was 2159.2 kcal/day. The baseline median value of GHGE was 5.8 kg CO_2_-eq/day, LU 4.5 m^2^*y/day, FEU 40.8 MJ/day, and *p*ReCipe 0.6 points in both groups. While contributing to 11.2 *E*%, total meat intake accounted for approximately 35.1% of daily diet-associated GHGE, 39.1% of LU and 21.2% FEU in the F-BA group at baseline, with similar contributions in the control group (Table 3). The impact of dairy on GHGE, fat on LU and fish and vegetables on FEU was substantial (dairy: 14.1% of GHGE; fat: 10.9% of LU; fish: 16.5% of FEU and vegetables: 15.1% of FEU). Sweets/extras contributed most to total caloric intake in both groups at baseline (19 *E*%), yet had a relatively low impact on GHGE (5.5%), LU (7.0%) and FEU (6.3%).

**Table 3:** Food group contributions to total caloric intake (*E*%) and to daily diet-associated greenhouse gas emissions (GHGE) (% of total kg CO_2_-eq/day), land use (LU) (% of total m^2^*y/day), and fossil energy use (FEU) (% of total MJ/day) in the food-based behavioral activation therapy (F-BA) group and control group at baseline

Food group	F-BA group (*N* = 373)				Control group (*N* = 371)			
	*E*%	GHGE	LU	FEU	*E*%	GHGE	LU	FEU
Vegetables	4.2	8.9	4.3	15.1	4.3	9.5	4.5	16.1
Fruit	7.3	5.3	6.7	6.6	7.4	5.4	6.8	6.6
Fish	3.4	9.6	1.6	16.5	3.2	8.9	1.4	15.3
Meat								
Red meat	8.7	30.0	31.8	15.9	9.3	30.5	33.0	16.6
Poultry	2.5	5.1	7.3	5.3	2.5	5.1	7.4	5.3
Egg/soy	2.5	1.3	2.3	1.7	2.7	1.4	2.5	1.9
Pulses/legumes	2.1	1.2	3.8	1.7	2.2	1.2	3.9	1.7
Nuts	1.3	0.2	1.0	0.3	1.4	0.3	0.9	0.3
Potatoes	1.7	0.6	0.5	0.8	1.6	0.5	0.4	0.7
Cereals								
Whole grains	8.4	2.3	4.7	2.4	8.5	2.4	5.0	2.5
Refined grains	9.0	3.0	2.5	3.6	8.8	2.9	2.4	3.4
Dairy								
Low-fat dairy	4.6	6.1	3.1	3.6	4.7	6.2	3.2	3.7
High-fat dairy	8.1	8.0	4.4	3.9	7.9	7.7	4.3	3.8
Fat								
Olive oil	4.7	0.6	7.8	0.4	4.2	0.5	6.9	0.3
Other fats/oils	5.2	1.9	3.1	1.3	5.0	1.8	3.0	1.2
Sweets/extras	19.0	5.5	7.0	6.3	18.9	5.3	6.8	6.2
Beverages								
Soft drinks	0.8	1.6	1.8	2.8	0.8	1.5	1.6	2.5
Alcoholic	2.5	2.4	2.4	3.3	2.5	2.5	2.4	3.4
Water/coffee/tea	3.4	5.9	3.4	8.1	3.5	5.9	3.2	8.0

### Changes in food intake and environmental impact of the diet during the intervention

**Fig. 1:**
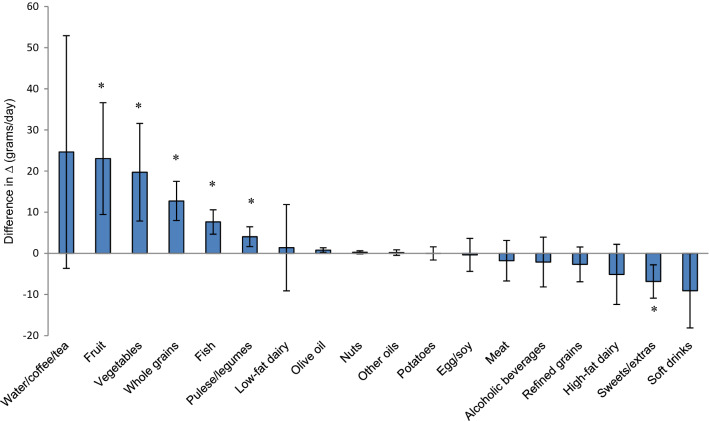
Effect of the food-related behavioral activation therapy (F-BA) intervention on intake of 18 food groups in overweight adults with subsyndromal symptoms for depression during the 12-month MooDFOOD depression prevention trial (*N* = 744). The bars represent the difference in change in intake from baseline to 12 months between participants who received F-BA intervention (F-BA group) and participants who did not receive F-BA intervention (control group) when controlling for baseline value of outcome, age, sex and site. The lines represent 95% confidence intervals. *Significant at Holm–Bonferroni-corrected *P* value

No difference in change in total caloric intake was apparent between the groups after 12 months (22.9 kcal/day; 95% CI − 10.1 to 55.9; *P *= 0.173). Significant increases in reported daily intake from T0 to T12 were observed for vegetables (19.7 g/day; 7.8–31.6; *P *= 0.001), fruit (23.0 g/day; 9.4–36.6; *P *= 0.001), fish (7.6 g/day; 4.6–10.6; *P *< 0.001), pulses/legumes (4.0 g/day; 1.6–6.5, *P *= 0.001), and whole grains (12.7 g/day; 8.0–17.5, *P *< 0.001), while a significant decrease was observed for sweets/extras (− 6.8 g/day; − 10.9 to − 2.8; *P *= 0.001) in the F-BA group relative to the control group (Fig. 1). Differences in change in reported intake of olive oil (0.8 g/day; 0.2–1.4, *P *= 0.006) and soft drinks (− 9.1 g/day; − 18.1 to − 0.1, *P *= 0.048) in the F-BA group relative to the control group were not significant after Holm–Bonferroni correction. No difference in change in reported meat consumption was evident, also when specifying red meat (− 3.4 g/day; − 6.7 to − 0.04; *P *= 0.047) and poultry (1.7 g/day; − 0.9 to 4.4; *P *= 0.197). The difference in change in red meat consumption was non-significant after Holm–Bonferroni correction.

These changes in food intake had no effect on diet-associated GHGE, LU or the *p*ReCiPe score, but led to a statistically significant 3.6% increase in FEU (1.6 MJ/day, 0.8–2.4, *P *< 0.001) in the F-BA group compared to the control group (Table 4). When the differences in change in environmental outcomes were controlled for total caloric intake, i.e., difference in change when energy intake would remain constant over the 12-month period between the F-BA group and the control group, the results were attenuated. However, the difference in change in FEU of the diet remained significant (1.2 MJ/day; 0.5–1.8; *P *< 0.001) and differences in change in GHGE, LU and *p*ReCiPe score remained insignificant.

**Table 4:** Effect of the food-related behavioral activation therapy (F-BA) intervention on environmental impact of diet in overweight adults with subsyndromal symptoms for depression during the 12-month MooDFOOD depression prevention trial (*N* = 744)

Environmental outcomes	*β*^a^	SE	95% CI	*P* value
GHGE^b^ (kg CO_2_-eq/day)				
Model 1^f^	0.060	0.060	− 0.058 to 0.179	0.320
Model 2^g^	0.004	0.045	− 0.084 to 0.092	0.933
LU^c^ (m^2^*y/day)				
Model 1	0.024	0.049	− 0.071 to 0.119	0.622
Model 2	− 0.017	0.035	− 0.084 to 0.051	0.630
FEU^d^ (MJ/day)				
Model 1	1.625	0.418	0.807 to 2.444	< 0.001*
Model 2	1.118	0.323	0.547 to 1.815	< 0.001*
*p*ReCiPe score^e^ (points)				
Model 1	0.008	0.006	− 0.003 to 0.019	0.173
Model 2	0.002	0.004	− 0.006 to 0.010	0.460

*Significant at Holm–Bonferroni-corrected *P* value

^a^Unstandardized beta coefficient of difference in change from baseline to 12 months between participants who received the F-BA intervention (F-BA group) and participants who did not receive the F-BA intervention (control group)

^b^Greenhouse gas emissions

^c^Land use

^d^Fossil energy use

^e^Weighted average of GHGE, LU and FEU

^f^Model 1 controls for baseline value of outcome, age, sex and site

^g^Model 2 is model 1 plus total caloric intake as a covariate

The difference in change in GHGE, LU, FEU, and *p*ReCiPe of each food group as well as the overall diet are shown in the Online Resource 1, Figs. 2–5. The increase in fish intake by the F-BA group relative to the control group contributed the most to the increasing effect of the F-BA intervention on diet-associated FEU (Online Resource 1, Fig. 4). The relative increase in intake of fish contributed to an increase in FEU by 1.2 MJ/day (0.7–1.6; *P *< 0.001), vegetables to an increase by 0.4 MJ/day (0.1–0.6; *P *= 0.008), fruit to an increase by 0.2 MJ/day (0.1–0.04; *P *< 0.001), whole grains to an increase by 0.1 MJ/day (0.1–0.2; *P *< 0.001), and pulses/legumes to an increase by 0.1 MJ/day (0.03–0.10; *P *= 0.001), while the relative decrease in intake of sweets/extras contributed to a decrease by 0.1 MJ/day (− 0.2 to − 0.1; *P *= 0.001) in the F-BA group compared to the control group during the intervention.

### Post hoc per protocol analysis results

In total, 365 out of 512 participants randomized to the F-BA intervention attended at least 8 therapy sessions and were considered compliant. Among those who attended at least 8 therapy sessions, 6 had missing dietary data at T0 and 45 had missing dietary data at both T6 and T12. In addition, 3 participants had over-reported energy intake at T0 and 1 participant at T6 and T12 were excluded from the analysis. Thus, 310 participants were included in the F-BA compliant subgroup. Indeed, the effect of the intervention was stronger among those who were most compliant to the F-BA intervention compared to the control group, i.e., a greater change in intake of the same food groups were observed compared to ITT analysis. Significant increases in reported daily intake from T0 to T12 were observed for vegetables (27.1 g/day; 14.9–39.3; *P *< 0.001), fruit (26.1 g/day; 12.0–40.2; *P *< 0.001), fish (9.9 g/day; 6.7–13.0; *P *< 0.001), pulses/legumes (5.2 g/day; 2.7–7.8, *P *< 0.001), and whole grains (14.8 g/day; 9.7–19.9, *P *< 0.001), while a significant decrease was observed for sweets/extras (− 7.5 g/day; − 11.7 to − 3.2; *P *= 0.001) in the F-BA subgroup relative to the control group. This in turn led to a statistically significant 4.8% increase in diet-associated FEU (2.2 MJ/day; 1.3–3.0; *P *< 0.001), and no change in GHGE, LU, and *p*ReCiPe score.

## Discussion

We found that the F-BA intervention led to changes in food intake among overweight adults with subsyndromal symptoms of depression according to the MooDFOOD dietary guidelines: significant increases in consumption were reported for some of the food groups promoted (i.e., vegetables, fruit, fish, pulses/legumes, and whole grains) and a significant decrease was reported for one of the food groups discouraged (i.e., sweets/extras) by the guidelines. The differences in change are roughly equivalent to eating an additional 3/4 tablespoon of mixed vegetables a day, 3/4 of an apple a day, 1/2 slice of whole grain bread a day, 1½ servings of salmon a month, and 3¼ tablespoons of legumes a month while refraining from eating about 2 teaspoons of sugar a day [52, 53]. However, these dietary improvements resulted in an unfavorable increased FEU of the overall diet equivalent to an additional 1.5 L of petrol a month [54], and no difference in change in diet-associated GHGE, LU or *p*ReCiPe score. Our results indicate that a shift towards a healthier Mediterranean-style diet does not necessarily reduce diet-associated environmental impact in a real-life setting.

Our findings are consistent with other studies that modeled hypothetical dietary changes towards a healthier diet and observed either no change or an increase in environmental impact of the healthy diet scenarios [40, 55–58]. Such studies have found that there is a greater need for increasing consumption of vegetables, fruit, legumes, and fish than decreasing consumption of meat and dairy products to achieve a healthy diet, resulting in a net-positive effect on environmental impact of the diet (i.e., higher environmental impact). Yet, when meat consumption is substantially reduced, then the environmental benefits of reducing meat consumption outweigh the increase in environmental impact due to increased intake of vegetables, fruit, legumes, and fish when shifting towards a recommended healthy diet [15, 19]. Although the MooDFOOD dietary guidelines recommended to limit meat intake to 300 g/week, which was substantially lower than the baseline median intake of 857 g/week in the F-BA group, the intervention did not lead to changes in meat intake. Because the current study observed the environmental impact of dietary changes in a real-life setting, it may inherently encounter constraints such as individual preferences, values, and personal efficacy not accounted for in the previous studies examining the environmental impact of hypothetical dietary change. Consumer behavior studies that have explored attitudes and intentions towards meat consumption have found that there is low willingness to change meat consumption behavior in terms of reducing or substituting meat in Europe [59, 60]. For many people meat holds an important place in the diet as it is associated with pleasure and various personal, social, and cultural-oriented values such as health and strength [60, 61]. Therefore, future dietary interventions should consider current values attached to meat and other constraints opposing changes in meat consumption to achieve healthy, sustainable diets.

We found that the increased impacts on GHGE and LU from the increased intake of fruit, vegetables, fish, pulses/legumes, and whole grains were collectively offset by the reduced impacts on GHGE and LU from the decreased intake of sweets/extras. Our results are in line with the weight loss trial which found that a reduction in intake of sweets, snacks, and soft drinks and an increase in intake of fruit and vegetables led to no change in overall carbon footprint of the diet [20]. However, we found that observed dietary change led to an increase in FEU of the overall diet, which may be attributable to the relative increase in fish, as fisheries are generally energy-intensive operations [62, 63]. An additional explanation for finding an increase in FEU of the overall diet may be due to the relative increase in vegetable consumption combined with the use of environmental data from the Netherlands, where the impact of vegetables on FEU is relatively high because of the use of greenhouses running on fossil energy [64]. Similar changes in FEU were found in two modeling studies, which found that switching from the current average American diet to a healthy diet recommended in the Dietary Guidelines for Americans would increase FEU, mainly caused by the recommendation to substantially increase the intake of fruits, vegetables and dairy products [57, 58]. Thus, while the increase in consumption of fruit, vegetables, fish, pulses/legumes, and whole grains may make a diet healthier, it may make it less sustainable unless replacing other food groups with similar or higher environmental impact, i.e., meat.

The actual change in the environmental impact of the diet is highly sensitive to the change in food choices, since there is very large variation in the GHGE, LU, and FEU levels per unit food within both the animal-based and plant-based food groups [11, 55]. To achieve healthy and sustainable diets, future dietary interventions must consider the environmental impact associated with different food groups (e.g., high-impact meat versus low-impact legumes), and also the environmental impact of various foods within food groups, such as beef (high impact) and poultry (lower impact) or tomatoes grown in a greenhouse (high impact) or in a field (lower impact) [55, 65]. As there are many different ways to follow the dietary guidelines provided by the MooDFOOD trial, different choices within food groups, for example, how to meet 300–400 g of vegetables per day, can lead to different environmental impacts. This was illustrated by Van Kamp and colleagues who found that compared to the current average Dutch diet, two healthy diets defined by the Dutch dietary recommendations resulted in either a 3% or 28% reduction in GHGE, with greater reductions in GHGE when dietary recommendations were met by including only foods with low impact on GHGE [55]. Furthermore, a reduction in overall caloric intake without changing the composition of the diet has been shown to result in lower environmental impact of the diet [16, 58]. Thus, for dietary guidelines to have a positive impact on the environment as well as health, consideration of the environmental impact of individual foods and food groups as well as total caloric intake is needed in addition to health considerations.

Our study has some limitations. First, FFQs are prone to recall bias and selective misreporting of consumption of certain foods [66]. In particular, the potential of differential response bias is high as exposure to the intervention itself can create differential error in reporting, with the treatment group possibly over-reporting foods promoted during the F-BA intervention (e.g., vegetables) and under-reporting foods that were discouraged (e.g., sweets) compared to the control group [67]. Second, the study population was overweight and at high risk of depression, limiting the generalizability of our findings to other populations. Third, the LCA data used to estimate the environmental impact of the diet comprised of a mix of data representative of an average Dutch diet as well as an average European diet. There are differences in geography, climate and production, processing, and distribution systems in Germany, UK, Spain and The Netherlands which may influence the actual environmental impact of diets in each country. Thus, while the LCA data used does not explicitly represent the production practices in each country, in the absence of country-specific data, these data serve as a proxy to provide a rough estimation on diet-level impacts. Fourth, while the LCA data sets used allowed us to study multiple environmental impact indicators, namely, GHGE, LU, and FEU, other important aspects such as water use, eutrophication, and biodiversity loss are missing in this analysis, because reliable data were not available. For instance, GHGE, LU, and FEU do not reflect the sustainability concerns of increasing fish consumption with regard to marine biodiversity loss and overfishing. Finally, the studied environmental indicators also have limitations. The LU indicator does not differentiate between different types and quality of land, which will bias livestock products to having higher impacts even if they graze on land unsuitable for cropping [2]. Furthermore, GHGE and FEU are strongly correlated (0.913, *P *< 0.001), as carbon dioxide emitted from fossil fuels used in the food chain directly contributes to GHGE of the diet [63]. Despite the considerable overlap between these two indicators, they measure different pressures, i.e., GHGE is a proxy for polluting emissions and FEU is a proxy for resource depletion [41, 68]. The strengths of this study include the use of an FFQ validated to measure food intake across different European countries [37], its large sample size compared to other dietary interventions looking at changes in food intake [69] and the use of three environmental impact indicators in addition to a weighted score measuring the overall environmental impact. Most importantly, the MooDFOOD trial allowed for the assessment of environmental impact of dietary change under real-life circumstances, while the previous studies have mainly measured the environmental impact of hypothetical dietary change.

## Conclusion

Our research shows that the food-related behavioral activation therapy led to favorable changes in food intake according to the Mediterranean-style dietary guidelines, but to no change in GHGE, LU or *p*ReCiPe score, and a small unfavorable change in FEU of the diet. To generate dietary change that is favorable for both health and the environment, dietary interventions must focus specifically on incorporating environmental sustainability aspects, in particular focusing on reducing and replacing meat consumption, choosing foods within a healthy diet that have low environmental impact and reducing total caloric intake. Furthermore, cultural, social and personal values around eating meat should be integrated. Future research should evaluate the environmental impact of dietary change in individuals who receive dietary guidelines especially designed to decrease the environmental impact of the diet and improve health, simultaneously.

## Electronic supplementary material

Below is the link to the electronic supplementary material.

Supplementary material 1 (DOCX 121 kb)

Supplementary material 2 (XLSX 39 kb)
