# Supplementary material for: Effect of food-related behavioral activation therapy on food intake and the environmental impact of the diet: results from the MooDFOOD prevention trial
Source: Eur J Nutr. 2019 Oct 23;59(6):2579–91. doi: 10.1007/s00394-019-02106-1 (PMC7413920; doi:10.1007/s00394-019-02106-1)
Supplement: Supplementary file 1 — Supplementary material 1 (DOCX 121 kb) [file 394_2019_2106_MOESM1_ESM.docx]

**SUPPLEMENTARY TABLES & FIGURES – ONLINE RESOURCE 1**

**TITLE**

Effect of food-related behavioral activation therapy on food intake and the environmental impact of the diet: Results from the MooDFOOD prevention trial

**JOURNAL**

European Journal of Nutrition

**AUTHORS AND AFFILIATIONS**

Alessandra C. Grasso^1,2*^, Margreet R. Olthof^1,2,^, Corné van Dooren^3^, Miquel Roca^4^, Margalida Gili^4^, Marjolein Visser^1,2^, Mieke Cabout^1,2^, Mariska Bot^2,5^, Brenda W. J. H. Penninx^2,5^, Gerard van Grootheest^2,5^, Elisabeth Kohls^6^, Ulrich Hegerl^7^, Matthew Owens^8^, Ed Watkins^8^, Ingeborg A. Brouwer^1,2^ & on behalf of the MooDFOOD Prevention Trial Investigators

*^1^ Department of Health Sciences, Faculty of Science, Vrije Universiteit (VU) Amsterdam, De Boelelaan 1105, 1081 HV Amsterdam, The Netherlands*

*^2^ Amsterdam Public Health Research Institute, Van der Boechorststraat 7, 1081 BT Amsterdam, The Netherlands*

*^3^ Netherlands Nutrition Centre (Voedingscentrum), Bezuidenhoutseweg 105, 2594 AC The Hague, The Netherland*

*^4^ Institut Universitari d’Investigació en Ciències de la Salut (IUNICS/IDISPA), Rediapp, University of Balearic Islands, Carretera De Valldemossa km 7.5, 07122 Palma de Mallorca, Spain*

*^5^ Department of Psychiatry, Amsterdam Universitair Medische Centra (UMC), VU Amsterdam, De Boelelaan 1117, 1081 HV Amsterdam, The Netherlands*

*^6^ Department of Psychiatry and Psychotherapy, Medical Faculty, University Leipzig, Semmelweisstr. 10, Haus 13, 04103 Leipzig, Germany*

*^7^ Department of Psychiatry, Psychosomatics and Psychotherapy, Medical Faculty, Goethe-University Frankfurt, Heinrich-Hoffmann-Str. 10, 60528 Frankfurt a.M., Germany*

*^8^ Department of Psychology, University of Exeter, Perry Road, Exeter EX4 4QG, United Kingdom*

**Corresponding author. Tel.: +31-20-59-84038, E-mail address:* [*alessandra.grasso@vu.nl*](mailto:alessandra.grasso@vu.nl)

| **Table 1** Adapted GA2LEN FFQ food sections & items used in MooDFOOD prevention trial with food group classification | |
| --- | --- |
| Food section/item | Food group classification |
| 1. **Bread and rolls** |  |
| 1. Any type of bread | - |
| 1. Wholemeal or brown bread (with or without seeds) | Whole grains |
| 1. White bread (e.g. baguette, rolls, sliced) | Refined grains |
| 1. Rye bread (any) | Whole grains |
| 1. Naan bread | Refined grains |
| 1. Chapatti | Refined grains |
| 1. Yeast based bread | Refined grains |
| 1. **Breakfast cereals** |  |
| 1. Any breakfast cereals | - |
| 1. Wheat germ | Whole grains |
| 1. Quaker (or other oat cereal) | Whole grains |
| 1. Corn-flakes | Refined grains |
| 1. All-bran cereals | Whole grains |
| 1. **Semolina** |  |
| 1. Couscous | Whole grains |
| 1. **Pasta (and wheat-derived foods)** |  |
| 1. Any pasta | - |
| 1. Plain (refined) pasta (e.g. spaghetti) | Refined grains |
| 1. Plain wholemeal (unrefined) pasta | Whole grains |
| 1. Filled pasta (with meat/cheese/vegetables) | Refined grains |
| 1. Noodles (excluding rice noodles) | Refined grains |
| 1. **Bakery products/desserts** |  |
| 1. Any cakes or pastries | - |
| 1. Cakes (e.g. sponge, chocolate) | Sweets/extras |
| 1. Pastries (e.g. croissants) | Sweets/extras |
| 1. Rolls (with/without filling) | Sweets/extras |
| 1. Muffins | Sweets/extras |
| 1. Doughnuts, buns (plain or filled) | Sweets/extras |
| 1. Rice pudding | Sweets/extras |
| 1. Cheesecake | Sweets/extras |
| 1. Pancakes | Sweets/extras |
| 1. Plain biscuits (with no fillings or cream) | Sweets/extras |
| 1. **Rice** |  |
| 1. Any rice | - |
| 1. White rice | Refined grains |
| 1. Brown/wholemeal (unrefined) rice | Whole grains |
| 1. Rice noodles | Refined grains |
| 1. **Sugar & jam** |  |
| 1. Table sugar (white) | Sweets/extras |
| 1. Jam | Sweets/extras |
| 1. Marmalade | Sweets/extras |
| 1. Honey | Sweets/extras |
| 1. **Sugar products excluding chocolate** |  |
| 1. Any sweets or bonbons | - |
| 1. Boiled sweets, toffees, caramels | Sweets/extras |
| 1. Mixed candies | Sweets/extras |
| 1. Cereal bars, flapjacks/fruit bar | Sweets/extras |
| 1. Ice lolly | Sweets/extras |
| 1. **Chocolate** |  |
| 1. Any chocolates | - |
| 1. Chocolate snack bars (e.g. Mars bar) | Sweets/extras |
| 1. Dark chocolate | Sweets/extras |
| 1. Milk chocolate | Sweets/extras |
| 1. **Vegetable oils** |  |
| 1. Any vegetable oil (blended) | - |
| 1. Sunflower oil | Other fats/oils |
| 1. Olive oil | Olive oil |
| 1. Extra virgin olive oil | Olive oil |
| 1. Palm oil | Other fats/oils |
| 1. **Margarine and fats of mixed origin** |  |
| 1. Any margarine or spread (excluding soya spread) | - |
| 1. Low-fat margarine | Other fats/oils |
| 1. Normal margarine | Other fats/oils |
| 1. Blended spreads | Other fats/oils |
| 1. Soya-based margarine or spreads | Other fats/oils |
| 1. Any margarines or vegetable spreads fortified with omega-3 | Other fats/oils |
| 1. **Butter and animal fats** |  |
| 1. Any butter | - |
| 1. Low/reduced fat butter | Other fats/oils |
| 1. Normal butter | Other fats/oils |
| 1. Lard | Other fats/oils |
| 1. **Nuts** |  |
| 1. Any nuts | - |
| 1. Peanuts | Pulses/legumes |
| 1. Cashew nuts | Nuts |
| 1. Almonds | Nuts |
| 1. Walnuts | Nuts |
| 1. **Legumes** |  |
| 1. Any legumes | - |
| 1. Kidney (red), black beans | Pulses/legumes |
| 1. Lentils | Pulses/legumes |
| 1. Chickpeas (also hummus) | Pulses/legumes |
| 1. Cluster beans (guar) | Pulses/legumes |
| 1. French beans (string beans) | Pulses/legumes |
| 1. Fava beans | Pulses/legumes |
| 1. Soya beans | Pulses/legumes |
| 1. **Vegetables excluding potatoes** |  |
| 1. Any vegetables (excluding potatoes) | - |
| 1. Lettuce | Vegetables |
| 1. Spinach (including lamb’s quarters) | Vegetables |
| 1. Chard | Vegetables |
| 1. Fenugreek | Vegetables |
| 1. Wild greens (e.g. watercress) | Vegetables |
| 1. Okra | Vegetables |
| 1. Tomato | Vegetables |
| 1. Aubergine | Vegetables |
| 1. Courgette | Vegetables |
| 1. Sweet peppers (e.g. red, green, yellow) | Vegetables |
| 1. Cucumber | Vegetables |
| 1. Bitter melon (Karela) | Vegetables |
| 1. Carrots | Vegetables |
| 1. Parsnip | Vegetables |
| 1. Turnip or Swede | Vegetables |
| 1. Artichoke | Vegetables |
| 1. Radish | Vegetables |
| 1. Beetroot | Vegetables |
| 1. Celery | Vegetables |
| 1. Coleslaw | Vegetables |
| 1. Sweetcorn | Vegetables |
| 1. Asparagus | Vegetables |
| 1. Herbs (e.g. mint, fennel, chive, basil, dill, coriander, parsley) | Vegetables |
| 1. Leek | Vegetables |
| 1. White/other mushrooms | Vegetables |
| 1. Onions | Vegetables |
| 1. Garlic | Vegetables |
| 1. Cauliflower | Vegetables |
| 1. Pumpkin | Vegetables |
| 1. Brussels sprouts | Vegetables |
| 1. Peas (green) | Pulses/legumes |
| 1. Broccoli | Vegetables |
| 1. Cabbage (e.g. white, green red, Savoy) | Vegetables |
| 1. Stuffed vegetables (e.g. vine/green leaves with rice or meat) | Vegetables |
| 1. Pickled vegetables (e.g. cucumber, radish, cabbage) | Vegetables |
| 1. Ginger (e.g. in savoury and sweet dishes, in infusion) | Vegetables |
| 1. **Starchy roots or potatoes** |  |
| 1. Any potatoes | - |
| 1. Mashed potatoes | Potatoes |
| 1. Baked/roasted/casserole | Potatoes |
| 1. Chips/French fries | Sweets/extras |
| 1. In salads | Potatoes |
| 1. Potato dumpling, bread dumpling, gnocchi | Potatoes |
| 1. Potato tortilla (omelette) | Potatoes |
| 1. Sweet potato | Potatoes |
| 1. **Fruits** |  |
| 1. Any fresh fruits | - |
| 1. Apple | Fruit |
| 1. Pear | Fruit |
| 1. Avocado | Fruit |
| 1. Mango | Fruit |
| 1. Apricot | Fruit |
| 1. Nectarine | Fruit |
| 1. Peach | Fruit |
| 1. Plum | Fruit |
| 1. Cherries | Fruit |
| 1. Rhubarb | Fruit |
| 1. Berries (e.g. blueberry, strawberry, blackcurrants, blackberry, raspberry) | Fruit |
| 1. Banana | Fruit |
| 1. Melon/ Watermelon | Fruit |
| 1. Grapes | Fruit |
| 1. Squeezed fresh fruit | Soft drinks |
| 1. Pineapple | Fruit |
| 1. Kiwi | Fruit |
| 1. Lemon | Fruit |
| 1. Orange | Fruit |
| 1. Mandarin/tangerine | Fruit |
| 1. Grapefruit | Fruit |
| 1. Tinned fruits | Fruit |
| 1. Raisins, sultana | Fruit |
| 1. Figs | Fruit |
| 1. Prunes | Fruit |
| 1. Olives (e.g. black, green) | Fruit |
| 1. Dates | Fruit |
| 1. **Fruit juices (1 glass 200 ml)** |  |
| 1. Concentrated juice, with sugar | Soft drinks |
| 1. Concentrated juice, without sugar (with sweetener) | Soft drinks |
| 1. **Non-alcoholic beverages (1 glass 200 ml)** |  |
| 1. Carbonated/soft drinks with sugar | Soft drinks |
| 1. Carbonated/soft drinks with artificial sweetener | Soft drinks |
| 1. Tap water | Water/coffee/tea |
| 1. Mineral water (e.g. still or sparkling) | Water/coffee/tea |
| 1. **Tea/coffee** |  |
| 1. Black tea (any) | Water/coffee/tea |
| 1. Coffee (instant or ground) | Water/coffee/tea |
| 1. Greek (Turkish) Coffee | Water/coffee/tea |
| 1. Green tea | Water/coffee/tea |
| 1. Peppermint tea | Water/coffee/tea |
| 1. Other herbal infusions | Water/coffee/tea |
| 1. **Beer (1/2 pint or 1 glass 200 ml)** |  |
| 1. Beer (any) | Alcoholic beverages |
| 1. **Wine (1 glass 125 ml)** |  |
| 1. Any wine | - |
| 1. Red wine | Alcoholic beverages |
| 1. White wine | Alcoholic beverages |
| 1. Rose wine | Alcoholic beverages |
| 1. **Other alcoholic beverages (1 glass 50 ml)** |  |
| 1. Fortified wines (Liqueurs) (e.g. Sherry, port, Madeira) | Alcoholic beverages |
| 1. Spirits (e.g. whisky, vodka, rum, gin) | Alcoholic beverages |
| 1. **Red meat and meat products** |  |
| 1. Any red meat (e.g. beef, veal, lamb, pork, game) | - |
| 1. Hot/cold roast beef, boiled beef, beef steak, fillet, loin | Meat |
| 1. Beef burger (hamburger) | Meat |
| 1. Minced beef meat (e.g chilli con carne, Bolognese sauce, meatballs) | Meat |
| 1. Beef meat in stew, casserole, in curry | Meat |
| 1. Pork cutlet, chop, steak, fillet, loin, pork ribs, minced | Meat |
| 1. Meat pies | Meat |
| 1. Sausages | Meat |
| 1. Veal | Meat |
| 1. Small game (e.g. rabbit, goat, pheasant, duck) | Meat |
| 1. Other game (e.g. deer, moose) | Meat |
| 1. Lamb (e.g. in stews, kebabs) | Meat |
| *Smoked/cured meat (3 slices)* |  |
| 1. Cured pork (cold or hot-cooked) | Meat |
| 1. Gammon, ham (e.g. Serrano, prosciutto) | Meat |
| 1. Dried cured sausages (chorizo, salchichon, salami) | Meat |
| 1. Frankfurter | Meat |
| 1. Bacon, bacon cubes | Meat |
| 1. Smoked lamb | Meat |
| 1. Smoked game (any) | Meat |
| 1. **Poultry** |  |
| 1. Any poultry with skin | - |
| 1. Any poultry without skin | - |
| *Fresh (unsmoked)* |  |
| 1. Chicken (e.g. boiled, roasted, chicken burgers) | Meat |
| 1. Chicken (e.g. stews or casserole) | Meat |
| 1. Turkey ( e.g. roasted, boiled, strips)   *Smoked or cured poultry* | Meat |
|  |  |
| 1. Any smoked/cured poultry | Meat |
| 1. **Offal** |  |
| 1. Liver (e.g. panita), pâtés, potted meat | Meat |
| 1. Other offal (e.g. tongue, brain, heart, kidney, tripe) | Meat |
| 1. **Fish and seafood** |  |
| 1. Any fish or seafood (fresh, tinned, smoked, etc) | - |
| 1. Fresh oily fish (e.g. salmon, tuna, trout, anchovy, herring, mackerel, sardine, gravalax, eel) | Fish |
| 1. Fresh white fish (e.g. hake/turbot, cod, haddock, plaice, whiting) | Fish |
| 1. Other fresh fish/seafood products (e.g. taramasalata) | Fish |
| 1. Fresh crustaceans and molluscs (e.g. mussel, crab, calamari, octopus, cuttlefish, shrimp, clam) | Fish |
| 1. Cured or smoked oily fish (e.g. sardines, tuna, salmon, kipper) | Fish |
| 1. Cured or smoked white fish (e.g. cod, bacalao, salt cod) | Fish |
| 1. Tinned fish (sardine, tuna or salmon) | Fish |
| 1. Tinned crustaceans and molluscs (e.g. mussel, crab, calamari, octopus, cuttlefish, shrimp, clam) | Fish |
| 1. **Eggs (from hen)** |  |
| 1. Any eggs | - |
| 1. Eggs (fried/poached/boiled/hard boiled/in sandwiches) | Egg/soy |
| 1. Egg-based savoury dishes | Egg/soy |
| 1. Egg-based desserts (e.g. egg cakes, tarts, egg and nut sweets) | Egg/soy |
| 1. **Milk, dairy and soya** |  |
| 1. Any milk (excluding soy) | - |
| *Cow milk* |  |
| 1. Full-fat milk | High-fat dairy |
| 1. Semi-skimmed milk | Low-fat dairy |
| 1. Skimmed milk | Low-fat dairy |
| 1. Milk fortified with omega 3 fatty acids | High-fat dairy |
| 1. Yogurt (any type including fromage) | High-fat dairy |
| *Soy* |  |
| 1. Soy milk | Egg/soy |
| 1. Yogurt from soy | Egg/soy |
| 1. Tofu | Egg/soy |
| 1. **Cheese** |  |
| 1. Any cheese | High-fat dairy |
| 1. Hard cheeses (e.g. Cheddar, parmesan) | High-fat dairy |
| 1. Soft cheeses (e.g. Brie, camembert, Philadelphia, tomini, boursault, brinza, chaource, coulommiers, Humboldt fog, kochkase) | High-fat dairy |
| 1. Semi-hard cheeses (e.g. Gouda, Emmental/Edam) | High-fat dairy |
| 1. Cottage cheese (cheese curd natural/with flavouring) | Low-fat dairy |
| 1. Hard and semi-hard Greek cheeses (e.g. Kaseri, kefalotiri, Grafiera, Kefalograviera, Ladotiri) | High-fat dairy |
| 1. Fresh cheeses (e.g. Feta, mozzarella) | Low-fat dairy |
| 1. **Other milk-derived products** |  |
| 1. Ice cream | Sweets/extras |
| 1. Single cream | High-fat dairy |
| 1. Crème fraîche | High-fat dairy |
| 1. Sour cream | High-fat dairy |
| 1. Double or clotted cream | High-fat dairy |
| 1. **Miscellaneous food** |  |
| 1. Dressing sauces (e.g. French, Cesar, thousand island) | Other fats/oils |
| 1. Mayonnaise | Other fats/oils |
| 1. White sauce | Sweets/extras |
| 1. Ketchup | Sweets/extras |
| 1. Instant soup | Sweets/extras |
| 1. Pizza (any) | Sweets/extras |
| 1. Brown sauce | Sweets/extras |

| **Table 2** Baseline characteristics of participants of the MooDFOOD prevention trial included in the mixed model analysis and excluded from analysis due to missing dietary data or implausible reported total caloric intake at T0 or at both T6 and T12 | | |
| --- | --- | --- |
| Characteristic | Included  n=744 | Excluded  n=281 |
| Sex^a^  Female  Male | 75.4 (561)  24.6 (183) | 75.1 (211)  24.9 (70) |
| Age (years)^b^ | 47.6 + 13.1 | 43.8 + 12.6 |
| Education^a^  Low  Middle  High | 9.7 (72)  47.2 (351)  43.1 (321) | 11.0 (31)  52.3 (147)  36.7 (103) |
| Site^a^  Germany  United Kingdom  Spain  The Netherlands | 30.4 (226)  24.7 (184)  21.4 (159)  23.5 (175) | 18.1 (51)  24.9 (70)  33.1 (93)  23.8 (67) |
| History of depression^a^  Yes  No | 32.3 (240)  67.7 (504) | 36.7 (103)  63.3 (178) |
| Pills^a^  Multi-nutrient  Placebo | 48.3 (359)  51.7 (385) | 54.4 (153)  45.6 (128) |
| BMI (kg/m^2^)^b^ | 31.2 + 4.0 | 31.8 + 4.0 |
| Total energy intake (kcal/day)^c^ | 2078.4 (1636.0; 2554.6) | 2218.6 (1761.1; 2848.9)^d^ |
| ^a^ Values displayed as percentage (frequency); ^b^ Values displayed as mean + sd; ^c^ Values displayed as median with interquartile range (25; 75^th^ percentile); ^d^ Total caloric intake of those who over-reported energy intake only (n=9) | | |

**Fig 1** Flow diagram of MooDFOOD depression trial participants included in the ITT analysis

Randomized (n=1,025)

Allocation

Analysis

Allocated to F-BA (F-BA group) (n=512)

- Allocated to multi-nutrient (n=256)
- Allocated to placebo (n=256)

Allocated to no F-BA (control group) (n=513)

- Allocated to multi-nutrient (n=256)
- Allocated to placebo (n=257)

Lost to follow-up (n=123)

Reasons:

- No time/not interested (n=37)
- Contact lost (n=34)
- Physical health reasons (n=18)
- Mental health reasons (n=8)
- Other (n=22)
- Unknown (n=4)

Missing FFQ (n=134)

- Missing FFQ at T0 (n=34)
- Missing FFQ at T6 and T12 (100)

Analyzed N=373

Excluded from analysis N=139

Missing FFQ (n=138)

- Missing FFQ at T0 (n=52)
- Missing FFQ at T6 and T12 (86)

Analyzed N=371

Excluded from analysis N=142

Implausible total caloric intake (n=5)

- Implausible caloric intake at T0 (4)
- Implausible caloric intake at T6 and T12 (1)

Implausible total caloric intake (n=4)

- Implausible caloric intake at T0 (4)
- Implausible caloric intake at T6 and T12 (0)

Lost to follow-up (n=123)

Reasons:

- No time/not interested (n=46)
- Contact lost (n=29)
- Physical health reasons (n=15)
- Mental health reasons (n=6)
- Other (n=27)
- Unknown (n=0)

**Fig 2** Effect of the food-related behavioral activation therapy (F-BA) intervention on diet-associated greenhouse gas emissions in overweight adults with subsyndromal symptoms for depression during the 12-month MooDFOOD depression prevention trial (N=744)

*

*

*

*

The blue bars represent the difference in change in greenhouse gas emissions (GHGE; kg CO_2_-eq/day) from baseline to 12 months between participants who received the F-BA intervention (F-BA group) and participants who did not receive the F-BA intervention (control group) when controlling for baseline value of outcome, age, sex and site. The lines represent 95% confidence intervals. *Significant at Holm-Bonferroni-corrected *P*-value

**Fig 3** Effect of the food-related behavioral activation therapy (F-BA) intervention on diet-associated land use (LU) in overweight adults with subsyndromal symptoms for depression during the 12-month MooDFOOD depression prevention trial (N=744)

*

*

*

*

The blue bars represent the difference in change in land use (LU; m^2^*a/day) from baseline to 12 months between participants who the F-BA intervention (F-BA group) and participants who did not receive the F-BA intervention (control group) when controlling for baseline value of outcome, age, sex and site. The lines represent 95% confidence intervals. *Significant at Holm-Bonferroni-corrected *P*-value

**Fig 4** Effect of the food-related behavioral activation therapy (F-BA) intervention on diet-associated fossil energy use (FEU) in overweight adults with subsyndromal symptoms for depression during the 12-month MooDFOOD depression prevention trial (N=744)

*

*

*

*

*

*

The blue bars represent the difference in change in fossil energy use (FEU; MJ/day) from baseline to 12 months between participants who received the F-BA intervention (F-BA group) and participants who did not receive the F-BA intervention (control group) when controlling for baseline value of outcome, age, sex and site. The lines represent 95% confidence intervals. *Significant at Holm-Bonferroni-corrected *P*-value

**Fig 5** Effect of the food-related behavioral activation therapy (F-BA) intervention on *p*ReCiPe score in overweight adults with subsyndromal symptoms for depression during the 12-month MooDFOOD depression prevention trial (N=744)

*

*

*

*

The blue bars represent the difference in change in *p*ReCiPe score (points) of diet from baseline to 12 months between participants who received the F-BA intervention (F-BA group) and participants who did not receive the F-BA intervention (control group) when controlling for baseline value of outcome, age, sex and site. The lines represent 95% confidence intervals. *Significant at Holm-Bonferroni-corrected *P*-value
